# Effect of six-week traditional resistance and functional training on functional performance in female netball players

**DOI:** 10.1186/s13102-022-00402-8

**Published:** 2022-01-15

**Authors:** Dimitrije Kovac, Zarko Krkeljas, Ranel Venter

**Affiliations:** 1 Department of Sports Science, Stellenbosch University, Suidwal Road, Coetzenburg, Stellenbosch, 7600 South Africa; 2 Duke Kunshan University, 8 Duke Avenue, Kunshan, 215316 Jiangsu China

**Keywords:** Functional training, Strength and conditioning, Sport performance, FMS®

## Abstract

**Background:**

Improving the quality of functional movements in athletes generally requires additional training targeting specific functional deficiencies. However, well-rounded, traditional strength and conditioning program should also improve player’s movement quality. Therefore, the primary aim of this study was to compare the effect of two different six-week interventions on the functional score of female netball players.

**Methods:**

In a randomized controlled study, players were divided into control and intervention group. Both groups completed identical six-week strength and conditioning program, with the intervention group also completing additional corrective exercises three sessions per week during the same period.

**Results:**

The FMS® score was significantly higher in the intervention group after 6-week program (f = 9.85, *p* = 0.004). However, the differences in total score may be attributed mainly to differences between groups in active straight leg raise (*p* = 0.004) and trunk stability push-up test (*p* = 0.02), as other individual tests demonstrated similar time and group effect.

**Conclusion:**

These results indicate that although FMS® based intervention may improve overall functional movement score, the athletes in both groups have demonstrated similar improvements in most of the individual tests. Hence, a well-rounded strength and conditioning program incorporating athlete-specific exercises based on limitations identified in the functional movement screen, may result in a balanced training strategy and reduce the need for supplementary functional training sessions.

## Background

Due to high incidence of non-contact lower limb injuries in female netball [1], there has been a significant focus on injury prevention strategies in research and practice. Previous studies suggest that integrating functional and/or corrective exercise as part of athletes’ strength and conditioning program may be an effective injury prevention strategy [2–5]. In general, strength and conditioning programs often focus on strength, power, agility and speed, and may overlook the importance of player’s functional limitations and ability to correctly execute basic functional movements [5, 6]. However, coaches and trainers may not have the practical capacity to add additional training sessions to their schedule, or subsequently may risk overtraining or under-preparing their athletes. Therefore, it would be of significant value to investigate the effect of adding corrective exercises to “standard” strength and conditioning programs on quality of functional movements in athletes.

One identifying characteristic of netball is the footwork rule, which stipulates that after catching the ball, landing foot must stay grounded, and may only move as a pivot. Hence, most of the lower limb injuries in netball occur during landing [1, 7, 8]. Furthermore, the nature of the sport results in muscle imbalance between quadriceps and hamstring groups, which often predisposes female athletes to injuries of the anterior crucial ligament [9]. Consequently, strong emphasis in netball training program should be placed on correction of functional muscle imbalance, improving motor control during multidirectional sprinting, and landing [10], and teaching players to perform locomotor, manipulative, and stabilizing actions^6^. Overlooking the importance of these functions predispose netball players to injury [1, 5, 6].

One of the widely used tools to assess quality of the functional movement in athletes is the functional movement screen (FMS**®**) [11]. Significant research has been committed to addressing the relationship between FMS**®** and athletic performance or injury rates with rather equivocal results. While FMS**®** may not be a good indicator of the physical readiness or player performance [12], the aim of FMS**®** is only to identify compensatory movement patterns that indicate movement dysfunction^6^ that may predispose athletes to injury [5, 13–15]. To improve the functional ability of players and correct identified movement dysfunctions, Cook [16] recommends exercise program specifically designed to improve the score on the functional movement screen.

Therefore, the purpose of our study is to examine the effect of FMS® intervention on functional capacity of high-level female athletes and compare the outcomes to the standard strength and conditioning program. We hypothesize that functional intervention will result in significantly greater improvements in functional capacity of athletes relative to the standard strength and conditioning program.

## Materials and methods

### Experimental design

Convenient sample of 40 elite university players volunteered for the study and signed an informed consent prior to participation. The study was approved by the University’s Ethics Committee (SU-HSD-001873) and was performed in accordance with the standards of Helsinki Declaration.

Players were randomly selected into control and intervention groups using Excel. Both groups participated in the identical strength and conditioning program, but corrective exercises were added to the intervention group three times per week. The FMS**®** score was measured before and after 6-week training cycle. Players were excluded from the study if they had sustained any musculoskeletal injury in the six weeks prior to testing, had an ACL injury in the previous 6 months, or if they were undergoing any rehabilitative protocol at the time of testing. From the 40 players who were initially tested, nine were excluded from the study either due to withdrawal from the team, or minor injuries that prevented full participation in the intervention or testing. The 31 remaining players completed the full study (Table 1).

**Table 1:** The participant anthropometric characteristics

	N = 31 (mean ± SD)	Control (n = 19) (mean ± SD)	Experimental (n = 12) (mean ± SD)	Sig, p
Age (yrs)	19.9 ± 1.5	19.8 ± 1.5	20.0 ± 1.5	0.71
Height (cm)	174.7 ± 6.5	175.6 ± 6.7	173.3 ± 6.3	0.35
Weight (kg)	70.0 ± 7.7	70.5 ± 8.1	69.3 ± 7.4	0.67

### Procedures

Functional movement screen was part of standard pre-season fitness assessment and was performed on the first day, prior to any other tests and without warm-up [6, 11].

Functional movement screen, individual program design and implementation were carried out by an FMS level-2 certified specialist. Procedures for the functional movement screen have been well described by previous studies [6, 11] and high interrater reliability has been reported for specialists who have completed the FMS programs. Considering both groups attended regular netball training all players were instructed not to change their daily routine so as not to affect the outcomes of the study. Similarly, the corrective sessions were scheduled in a way that limits impact of any other potential confounding factors on the effectiveness on the sessions or the effort of the participants during the sessions.

Based on athletes’ initial FMS score and movement limitations identified by the functional screen, 6-week individual functional exercise program was designed according to the standardized corrective algorithm suggested by Cook [16]. The program progressed through three stages of functionality (Table 2) and were designed to correct dysfunctional movement patterns by focusing on mobility, static and dynamic stability. For example, a player with score of one (1) on ASLR and SM tests started with mobility exercises, whereas a player who scored one (1) on the TSPU test started with stability exercises 18. Players performed three sessions per week (between 30 and 40 min long) prior to each regular netball training session.

**Table 2:** Stages and progression of the intervention program

**Mobility**	
Stage 1	Active straight leg raise with core activation—4 sets, 6 repetitions
	Leg lowering 1–4 sets, 6 repetitions
	Hip flexor stretch—4 sets, 6 repetitions, 10 s hold
Stage 2	Leg lowering 2–4 sets, 6 repetitions
	Leg lock bridge—4 sets, 6 repetitions
	Deadlift patterning—4 sets, 6 repetitions
Stage 3	Leg lowering 2–4 sets, 6 repetitions
	Straight leg bridge—4 sets, 6 repetitions
	Single-leg deadlift patterning RNT—4 sets, 6 repetitions
**Stability**	
Stage	Quadruped core activation—4 sets, 6 repetitions
	Plank with knee flexion—4 sets, 6 repetitions
	Rolling pattern—4 sets, 6 repetitions
Stage 2	Hard roll—4 sets, 6 repetitions
	Plank with leg extension—4 sets, 6 repetitions
	Elevated push-up—4 sets, 6 repetitions
Stage 3	Push-up walk out—4 sets, 6 repetitions
	Half Turkish get up—4 sets, 6 repetitions
	Push-up—4 sets, 6 repetitions

Statistical analyses were completed using Statistica v.13 (Dell Inc., Round Rock, TX, USA). All data were analyzed for normality with a Shapiro – Wilk test. A one-way ANOVA was used to assess the differences between groups and between pre- and post-test results. Any significant differences were analyzed with LSD-post hoc test. Significance for all tests was set at *p* < 0.05.

## Results

There were no significant differences in FMS**®** score between groups before intervention (Table 3).

**Table 3:** Differences in pre-intervention functional movement scores

	Control (mean ± SD)	Experimental (mean ± SD)	Sig, p
Deep squat	1.6 ± 0.5	1.7 ± 0.4	0.35
Hurdle step	1.8 ± 0.7	1.6 ± 0.7	0.31
In-line lunge	1.8 ± 0.4	1.7 ± 0.4	0.81
Shoulder mobility	2.6 ± 0.5	2.8 ± 0.6	0.31
Active straight leg raise	2.1 ± 0.9	1.7 ± 1.0	0.22
Trunk stability push-up	1.6 ± 0.8	1.3 ± 0.8	0.33
Rotary stability	1.9 ± 0.2	2.0 ± 0.0	0.44
Total FMS score	13.7 ± 2.4	13.0 ± 1.6	0.40

**Fig. 1:**
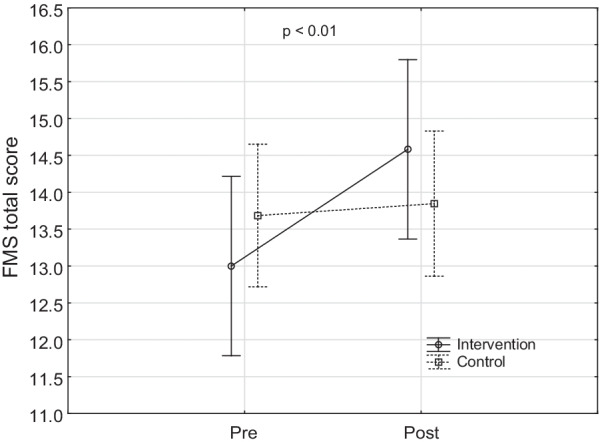
The differences in total functional movement score between groups after 6-week intervention with 95% confidence interval

The results show a significant main time-group effect for the FMS**®** score (f = 9.85, *p* = 0.004) (Fig. 1). While there was a significant improvement in score of the intervention group after intervention (f = 14.84, *p* = 0.0006), there was no significant improvement for players only participating in standard strength and conditioning program.

**Fig. 2:**
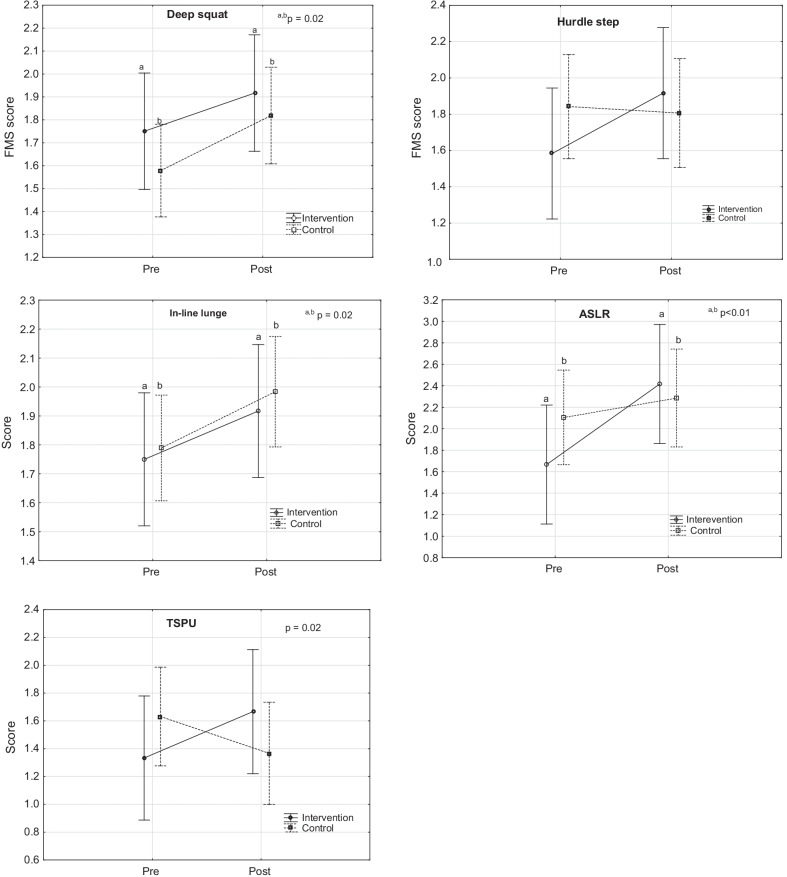
Differences in the individual FMS® tests between groups, pre- and post-intervention

Of the individual FMS**®** tests (Fig. 2) deep squat (f = 6.66, *p* = 0.01), in-line lunge (f = 5.90, *p* = 0.02) and active straight leg raise (f = 9.90, *p* = 0.004) significantly improved for both groups. Only group-time effect was noted for the trunk stability push-up test (TSPU) (f = 5.63, *p* = 0.02). Shoulder mobility and rotary trunk stability tests were scored 2 during pre- and post-testing and therefore was not depicted in graphs.

Relative to bilateral symmetry, there were no difference between groups for any test assessed for left and right side. However, overall participants scored significantly higher on their dominant side (right) in pre-testing during in line lunge (*p* = 0.008), shoulder mobility (*p* = 0.01), and potential differences were noted in hurdle step (*p* = 0.06) although statically not significant. Active straight leg raise was not different between left and right side (*p* = 0.22).

Relative to hurdle step, the results show a significant change in asymmetry between groups relative to pre- and post-post testing (f = 6.47, *p* = 0.017). LSD post-hoc indicates that there was no asymmetry between left and right side in the intervention group in post testing, with significant differences between left and right side in the control group during post testing (*p* = 0.04). Relative to in-line lunge, although the noted effect for asymmetry relative to group and period of testing was not statistically significant (f = 3.16, *p* = 0.09), differences between left and right side in pre-testing for intervention group (*p* = 0.04), were noted in post-testing (*p* = 0.05). On the other hand, pre-intervention differences for control group (*p* = 0.01), were not present in post-testing (*p* = 0.59). No significant effect or changes in asymmetry were noted for shoulder mobility and ASLR test.

## Discussion

The primary findings of this study indicate that 6-week corrective exercise intervention improved the overall FMS**®** score of the female netball players. However, the magnitude of change and the lack of changes in most of the individual tests, demand caution when interpreting these results. To the author’s knowledge, this is the first study to demonstrate these effects using intervention and control groups in netball.

When interpreting the effect of the intervention, consideration should be given to the age and gender of the population, type of sport or physical activity, and type and length of the intervention. In this study, we incorporated FMS**®** recommended intervention with the standard strength and conditioning program and found that adding corrective exercises partially improves the functional movements relative to strength program alone. In addition to the significant increase in an overall score, number of athletes scoring above 14 in the intervention group also significantly increased—a score identified to arguably predispose athletes to injury [6, 17]. These improvements are in agreement with the few studies that examined the effect of intervention on FMS**®** score, although on male mixed martial artists [5] and predominantly male firefighters [18].

However, the increase in score of the intervention group can be attributed only to changes in ASLR, TSPU and potentially the hurdle step scores of the intervention groups, as shoulder mobility, in line lunge, deep squat and rotary stability did not differ between groups. These results are somewhat expected as FMS**®** suggested intervention based on the initial assessment is largely based on improving core stability and control [16], while tests for which overall upper and lower body strength is required, have improved the same over the course 6-week for both groups. Hence, including few core corrective exercise into a “standard” strength and conditioning program might have a similar effect as including additional training with focus on FMS**®** corrective exercises.

Another component of improvement to consider when interpreting these results is the magnitude of improvement in the FMS**®** score. In this study, both groups were within 1 point from the injury cut of score of 14 in pre-testing, hence improvement by one point in any of the tests would shift players from being at risk of injury to less predisposed to injury. Consequently, although the number of players above the injury factor of 14 increased in the intervention group, the mean score was 14.5, an improvement of only 1.5 points. The size of improvement is comparable to other studies, but differences in sample population, type and length of the intervention should be considered during comparison. For example, in a study by Bodden et al. [5] male mixed martial arts athletes improved about 2 points after 4-week intervention, while Kiesel et al. [17] show 3 point improvement after 7 week intervention on professional football players. Additionally, Frost et al. [19] showed an improvement of only 0.4 points on total FMS**®** score on two different 12-week intervention programs on firefighters. Both Bodden et al. [5] and Kiesel et al. [17] studies conducted 4 sessions per week, while Frost et al. [19] used 3 sessions per week, similarly to this study. These results indicate potential confounding factors that extend beyond the differences in population and intervention methodology. Hence, volume and number of sessions during intervention is also highly individualized and may not necessarily reflect on the improvement in the FMS**®** score. Consequently, to ensure functional improvement in their athletes we suggest that trainers and coaches incorporate functional exercises at least 4 times per week, either as part of the general strength and conditioning training, or independent functional training sessions.

Previous studies have suggested that scoring criteria in FMS**®** could be amended to account for these scoring limitations, as a midrange score of 2 for example, may be given to an athlete with a wide range of movement limitations or patterns [5, 19]. In this study, this is evident from the shoulder and rotary stability tests, as all athletes scored 2 in pre and post testing even though some players might have improved the degree of compensation. This kind of scoring also does not accurately reflect athletes developing sport-specific characteristics that do not necessarily indicate predisposition to injury or are not relevant to the specific sport (e.g., leg strength asymmetry in soccer, reduced mobility of weightlifters or American football players). This may be the reason why several studies found limitations in using FMS**®** to identify movement deficiencies related to athletic performance in male [10] and female athletes [20], or general performance tests [21] including core stability [22].

## Conclusion

Functional movement program based on FMS® assessment and intervention design, may improve athletes’ functional movement ability. However, traditional strength and conditioning program has resulted in similar improvements for most of the individual tests, without the need for additional training sessions. Hence, we recommend the use of FMS® only as an assessment tool based on which coaches and trainers may design a well-rounded strength and conditioning program integrating athlete-specific functional exercises. This strategy will reduce the need for additional training sessions and prevent potential overtraining or under-recovery of athletes.

## Data Availability

The datasets used and/or analyzed during the current study are available from the corresponding author on reasonable request.

## Abbreviations

FMS: Functional movement screen
ACL: Anterior cruciate ligament
ASLR: Active straight leg raise
SM: Shoulder mobility
TSPU: Trunk stability push-up

## References

[1] Steele JR (1990). Biomechanical factors affecting performance in netball. Implications for improving performance and injury reduction. Sport Med.

[2] Benjaminse A, Otten E (2011). ACL injury prevention, more effective with a different way of motor learning?. Knee Surg Sport Traumatol Arthrosc.

[3] Benjaminse A, Welling W, Otten B (2015). Novel methods of instruction in ACL injury prevention programs, a systematic review. Phys Ther Sport.

[4] Elphinston J, Hardman SL (2006). Effect of an integrated functional stability program on injury rates in an international netball squad. J Sci Med Sport.

[5] Bodden JG, Needham RA, Chockalingam N (2015). The effect of an intervention program on functional movement screen test scores in mixed martial arts athletes. J Strength Cond Res.

[6] Cook G, Burton L, Hoogenboom B (2006). Pre-participation screening: the use of fundamental movements as an assessment of function—part 2. N Am J Sports Phys Ther.

[7] Hopper DM, Mcnair P, Elliott BC (1999). Landing in netball: effects of taping and bracing the ankle. Br J Sports Med.

[8] Otago L (2004). Kinetic analysis of landings in netball: is a footwork rule change required to decrease ACL injuries?. J Sci Med Sport.

[9] Myer GD, Ford KR, Foss KDB (2009). The relationship of hamstrings and quadriceps strength to anterior cruciate ligament injury in female athletes. Clin J Sport Med.

[10] Lockie RG, Schultz AB, Jordan CA (2015). Can selected functional movement screen assessments be used to identify movement deficiencies that could affect multidirectional speed and jump performance?. J Strength Cond Res.

[11] Cook G, Burton L, Hoogenboom B (2006). Pre-participation screening: The use of fundamental movements as an assessment of function–Part 1. North Am J Sport Phys Ther NAJSPT.

[12] Venter RE, Masterson C, Tidbury GB (2017). Relationship between functional movement screening and performance tests in elite university female netball players. South African J Res Sport Phys Educ Recreat.

[13] Chorba RS, Chorba DJ, Bouillon LE (2010). Use of a functional movement screening tool to determine injury risk in female collegiate athletes. N Am J Sports Phys Ther.

[14] Shojaedin SS, Letafatkar A, Hadadnezhad M (2014). Relationship between functional movement screening score and history of injury and identifying the predictive value of the FMS for injury. Int J Inj Contr Saf Promot.

[15] Bardenett SM, Micca JJ, DeNoyelles JT (2015). Functional movement screen normative values and validity in high school athletes: Can the FMS^TM^ be used as a predictor of injury?. Int J Sports Phys Ther.

[16] Cook G (2011). Movement: functional movement systems—screening, assessment, corrective strategies.

[17] Kiesel K, Plisky P, Butler R (2011). Functional movement test scores improve following a standardized off-season intervention program in professional football players. Scand J Med Sci Sports.

[18] Peate W, Bates G, Lunda K (2007). Core strength: A new model for injury prediction and prevention. J Occup Med Toxicol.

[19] Frost DA, Beach TAC, Callaghan JP (2012). Using the functional movement screen to evaluate the effectiveness of training. J Strength Cond Res.

[20] Lockie RG, Schultz AB, Callaghan SJ (2015). A preliminary investigation into the relationship between functional movement screen scores and athletic physical performance in female team sport athletes. Biol Sport.

[21] Parchmann CJ, McBride JM (2011). Relationship between functional movement screen and athletic performance. J Strength Cond Res.

[22] Okada T, Huxel KC, Nesser TW (2011). Relationship between core stability, functional movement and performance. J Strength Cond Res.

